# Legumes: A Vehicle for Transition to Sustainability

**DOI:** 10.3390/nu16010098

**Published:** 2023-12-27

**Authors:** Amalia E. Yanni, Sofia Iakovidi, Eleni Vasilikopoulou, Vaios T. Karathanos

**Affiliations:** Laboratory of Chemistry-Biochemistry-Physical Chemistry of Foods, Department of Nutrition and Dietetics, Harokopio University, 70 El. Venizelou Ave, 176-71 Athens, Greece; sophiaiakovidi@gmail.com (S.I.); elenavasilikopoulou@gmail.com (E.V.); vkarath@hua.gr (V.T.K.)

**Keywords:** legumes, pulses, health sustainability, environmental sustainability, greenhouse gas emissions, legume-based alternatives to animal products

## Abstract

Legumes are an excellent source of protein and have been used in the human diet for centuries. Consumption of legumes has been linked to several health benefits, including a lower risk of cardiovascular diseases, type 2 diabetes mellitus, and certain types of cancer, while legumes’ high fiber content promotes digestive health. Aside from the positive health benefits, one of the most significant advantages of legumes is the low environmental footprint of their cultivation. They can be grown in a variety of climates and soil types, and they require less water and fertilizer than other crops, making them a sustainable option for farmers. Thanks to their nutritional and physicochemical properties, they are widely used by the food industry since the growing popularity of plant-based diets and the increasing demand for alternatives to meat offers the opportunity to develop legume-based meat substitutes. As the use of legumes as a source of protein becomes widespread, new market opportunities could be created for farmers and food industries, while the reduction in healthcare costs could have a potential economic impact. Achieving widespread adoption of legumes as a sustainable source of protein requires coordinated efforts by individuals, governments, and the private sector. The objective of this narrative review is to present the benefits coming from legume consumption in terms of health and environmental sustainability, and underline the importance of promoting their inclusion in the daily dietary pattern as well as their use as functional ingredients and plant-based alternatives to animal products.

## 1. Introduction

The world population is predicted to reach 10.4 billion in 2100 [1], which will lead to an increase in global food demand. Nowadays, there is sufficient evidence that contemporary global food systems and consumption patterns are not sustainable for both human and planetary health [2].

Food systems are responsible for 21–37% of greenhouse gas (GHG) emissions, with livestock being the largest source [3]. Livestock production generates significant amounts of the three main GHGs (CO_2_, CH_4_, N_2_O) and is responsible for 14.5% of total anthropogenic GHG, with food systems contributing to at least one-third or more of global GHGs [4]. Meat production is the most significant source of CH_4_ emissions. More specifically, ruminants emit the highest GHG content per g of protein and kcal [5]. There is a large difference in GHG emissions between animal-based and plant-based foods. It has been reported that ruminant meat (beef and lamp) emissions per gram of protein are about 250 times higher than those of legumes [6]. Life cycle assessment studies have shown that pork, chicken, and seafood produce less GHG than beef. Nevertheless, even the animal products with the lowest impacts exceed the average GHG emissions of plant products [7].

Furthermore, per capita emissions from food consumption are much higher in countries with a very high human development index (HDI) than in countries with a high HDI and countries with a low HDI. At the same time, the mortality rate associated with red meat is almost nine times higher in countries with a very high HDI than in countries with a low HDI [8]. It is now widely considered that plant-based diets (PBDs) have a positive impact on the environment and health, and many advantages have been demonstrated, such as safety for human consumption, waste management, storage options, and lower GHG emissions compared to animal diets [5]. Although proteins from plant sources are considered to be of lower quality than proteins of animal origin, a well-designed plant-based diet can be both nutritionally adequate and environmentally sustainable [9].

Legumes are an excellent source of protein and have been used in the human diet for centuries. In recent times, legumes have drawn attention not only for their main role as an ecologically sustainable food source that is rich in protein, but also for their health benefits regarding multiple chronic non-communicable diseases. They have well-documented health effects when consumed as a part of many dietary patterns for healthy eating and disease management, such as the Mediterranean diet [10,11,12,13], Dietary Approaches to Stop Hypertension (DASH) [14], low glycemic index (GI) diets [15,16], and high fiber diets [17,18]. They are also an important component in many national dietary recommendations [United States Department of Agriculture (USDA) and Health and Human Services (HHS)] [19], the Prolepsis Institute recommendations [20], the National Health Service (NHS) Eatwell guide [21], as well as in recommendations for cancer prevention [American Institute for Cancer Research (AICR) [22] and World Cancer Research Fund International (WCRFI)] [23].

Although legumes have contributed to the human diet for over 60,000 years [24], recognition of their value has faded over the years [25], mainly because farmers have turned more towards cereals, and because the evolution of meat production has gradually changed traditional dietary patterns. Even in Mediterranean countries, where the populations with the highest legume consumption in Europe were located, meat consumption has largely replaced legume consumption [26]. According to the Global Dietary Database (GDD), global bean and legume consumption is lower than the suggested targets of 50 g/day; while high variability was noticed, it can be attributed to different cultural dietary patterns. In line with the GDD, Europe had by far the lowest consumption of legumes worldwide with intakes less than 10 g/day for more than one-third of countries. In Asia and the Pacific, 65% of countries fell below 50 g/day, while no country in Africa, North America, nor most countries in the Near East met the suggested daily target [27]. To highlight the role of pulses in healthy diets and their contribution to soil health and the environment, the United Nations General Assembly declared 2016 as the International Year of Pulses [28].

The aim of this narrative review is to present the benefits coming from legumes’ consumption and subsequent partial meat replacement, therefore increasing good quality life expectancy and leading to an important cutback in GHG. Promoting the use of legumes constitutes an urgent need to make food systems more sustainable and nutrition-sensitive. The lower intake of food of animal origin, and consequently, lower livestock production, would allow the conversion of feed crops into human food, and would not jeopardize food security in long term and heath sustainability. This would lead to better management of natural resources, as agro-ecosystems with plant-based proteins require far fewer resources and less energy input, while having healthier and more sustainable diets [29].

## 2. Methodology

The article is intended to present a broad scope of the theme, and a narrative approach was chosen as more flexible compared to a systematic review where the restrictive framework would not allow a wider exploration. The records used in this study were retrieved through Pubmed, Scopus, and Sciencedirect databases. The research was conducted until August 2023, and focused on articles published from 2010 and onwards, with exceptions for some widely acknowledged pieces. The keywords which were used and mirror the key concepts included: “legumes”, “pulses”, “health benefits”, “greenhouse gas emissions”, “plant-based alternatives”, “sustainability”, and other related terms in various combinations in order to permit selection of the related articles and also eliminate those that were not relevant. Exclusion criteria were articles regarding in vitro studies, animal studies, studies in pediatric populations and pregnant women, and articles regarding environmental attitudes and economic impact. Furthermore, those for which the full text was not available or were not written in the English language were excluded. Additional articles were identified by manually searching through reference lists of various publications. As for the methodology regarding the topics analyzed, these were formulated according to the results yielded from database research. Authors tried to include studies (research or review papers) with the best contribution in the field, integrating more recent publications in order to present the current knowledge.

## 3. Legumes and Health Sustainability

Legumes belong to the botanical family of Fabaceae (Leguminosae), and those which are consumed by humans are categorized into oilseed legumes (soybeans and peanuts) and non-oilseed legumes. The non-oilseed legumes are further categorized into pulses (chickpeas, cowpeas, dry beans, dry peas, lentils)—which are the dried, mature seeds of the pods—and undried legumes (snap beans and snap peas)—which are harvested before drying and may be consumed with or without their pods [30]. The nutritional profile of legumes varies between the different categories [31]. The main nutritional characteristics of the most common consumed legumes are presented in Table 1. It is evident from the nutritional content of the different types of legumes that oil seed legumes, namely soybeans and peanuts, have a higher protein and total fat content as well as a lower carbohydrate content when compared to non-oil seed legumes. Due to these differences in their characteristics, peanuts were not included in this report, and soybeans were seldom incorporated.

Extensive research has been carried out regarding the beneficial properties of pulses when it comes to their nutritional composition. They are characterized by low energy density, low GI, high fiber, and high protein content. They are also low in total fat, high in monounsaturated fatty acids (MUFA), polyunsaturated fatty acids (PUFA), plant sterols, vitamins, and minerals [32]. It is consistently documented in the literature that their protein, fiber, mineral and phytochemical contents especially may be the key factor that contributes to their health effects, mainly the metabolic ones [32,33,34]. Nevertheless, the exact nutritional value of legumes is affected by multiple factors. Most of their characteristics are affected by the specific type of legume and the cultivar. Processing methods such as soaking, boiling, microwaving, and autoclaving do not significantly alter the protein content, but can increase protein and starch digestibility and in some instances also increase the lipid and fiber content. The process of germination has been found to increase legumes’ mineral, vitamin B complex, and antioxidant contents [35]. A publication assessing the effect of industrial-scale germination on green-pea and chickpea nutritional values reported an increase in available carbohydrates regarding peas, and a higher protein content in both. However, the expected increase in antioxidants and minerals was not observed [36]. Furthermore, leguminous seeds have a high content of anti-nutrients and non-nutritive compounds, which hinder to some extent the absorption of vitamins and other nutrients. Anti-nutrients which are found in legumes include phytic acid, saponins, polyphenols, and alpha-amylase inhibitors, among others [37]. Phytic acid lowers the absorption of zinc, iron, calcium, magnesium, and copper, and can reduce the nutritional value of protein [38]. Tannins can also decrease the absorption of minerals and have been suggested by research as contributing to iron-deficiency anemia [39].

However, the content of anti-nutrients differs depending on the specific type of legume and the various methods of cooking and processing. Phytic acid is found at levels from 0.4−6.4% by weight. The content of saponins, which are mainly provided to the human diet by soyabeans, ranges between 0.5−5% dw. The levels of polyphenols vary greatly even between cultivars of the same species since parameters like light, germination, variety, processing, and storage affect their levels. The content in legumes ranges between 34–1710 mg/100 g dw [40].

The amounts of anti-nutrients in specific legumes, as well as the effects of different preparations on their content, can be found in the detailed review by Petroski and Minich [41]. Anti-nutrients may be reduced or eliminated through physical and chemical procedures such as soaking, cooking, germination, fermentation, selective extraction, irradiation, enzymic treatment, or a combination of them [37]. It is known that soaking before cooking reduces cooking time because it facilitates gelatinization and protein denaturation, resulting in softening of the texture. Soaking in simple water seems to not reduce the tannin content of legumes; however, the addition of sodium bicarbonate eliminates tannins and reduces trypsin inhibitor activity [42].

On the other hand, these same compounds have beneficial effects for humans. For example, isoflavones, which are phytoestrogens mainly in soybeans and chickpeas, have antioxidant and estrogenic effects, and thus are useful in the prevention of cardiovascular diseases [33,43]. In addition, phytic acid has an antioxidant capacity and therefore protects against DNA damage [41]. Tannins possess antioxidant, anticarcinogenic, immunomodulatory, and cardioprotective actions in humans [41]. Bioactive peptides inhibit the function of enzymes such as glucosidase and α-amylase, thus obstructing carbohydrate digestion, which is useful in the management of insulin-resistant and type 2 diabetes mellitus (T2DM) patients [16]. Generally, daily amounts of 150 g of cooked pulses seem to be enough to reap their benefits in all areas of health [32].

### 3.1. Oxidative Stress and Inflammation

To start with, reductions in oxidative stress and pro-inflammatory markers are thought to contribute to the overall health benefits exhibited from a legume-rich diet. In a review paper published in 2023 by Grdeń et al. [16], the authors concluded that non-soy legumes can lower C-reactive protein (CRP) blood levels, and they also described multiple anti-radical activities. A cross-over randomized controlled trial with a duration of 8 weeks and a wash-out period of 4 weeks in 31 overweight T2DM patients found significant reductions concerning CRP, interleukine-6 (IL-6), and tumor necrosis factor-a (TNF-a). These anti-inflammatory and antioxidant properties are thought to stem from some of the legumes’ aforementioned bioactive compounds (namely phytic acid and phenolic compounds i.e., tannins, anthocyanins, and other flavonoids), high magnesium and dietary fiber content, as well as them substituting animal protein sources [44,45].

### 3.2. Overweight and Obesity

Overweight and obesity are public health problems with a high prevalence to this day. Current literature confirms that legumes can contribute to the weight management and prevention of obesity. A study using data from the National Health and Nutrition Examination Survey (NHANES) reported that participants who consumed beans had lower body weight, smaller waist circumference, and lower body mass index (BMI) when compared to non-consumers. Also, a 22% less likelihood of becoming obese in the future was noted [46]. A systematic review and meta-analysis of 21 randomized clinical trials (RCTs) with a duration of at least 6 weeks concluded that intervention diets with pulses (mean content 132 g per day) led to greater weight loss compared to diets without pulses. This effect was statistically significant regarding both hypocaloric diets and diets without any energy restrictions. No statistically significant relationship with waist circumference was identified, but there was a trend of greater body fat loss with pulse-containing diets [47]. Significantly larger reductions both in body weight and waist circumference for obese and overweight individuals were reported when comparing hypocaloric diets rich in non-oilseed legumes (average of 150 g/day, cooked) with control hypocaloric diets such as high-protein or low-carb diets. This was mostly attributed to the high fiber content in legume-rich diets. A rise in mitochondrial oxidation markers observed in such diets suggests another possible physiological mechanism for the greater weight loss observed [32]. The high fiber content of legumes has been widely acknowledged as one of the main reasons why they contribute to weight management, either by reducing energy intake or by increasing satiety [34,46]. Increased feelings of satiety have been very well documented in the literature when it comes to legume consumption, and have also been attributed to their high protein content, apart from fiber [34,46,48,49,50]. Improved satiety can help the individual better control their eating habits, and therefore adhere more easily to nutrition recommendations benefiting both their weight and health status. Another possible reason for the increased satiation reported in the literature could be legumes’ low GI [46,47,48,49,50]. It has also been proposed that bioactive peptides found in soy and fermented bean seeds can inhibit pancreatic lipase, therefore reducing fat absorption of the meal that contains them [32].

It has to be mentioned that the studies included in this section of the manuscript did not focus on comparing the effects of oilseed versus non-oilseed legumes. This could be a very interesting prospect for future research.

### 3.3. Cardiovascular Diseases

Cardiovascular diseases (CVD) are the primary cause of mortality worldwide [51]. Research focusing on the effect of legume consumption on the cardiovascular system includes many studies on primary prevention by means of regulating CVD risk factors. These mainly entail hypertension, hyperlipidemia, diabetes, and obesity. The effect of legumes on weight management has already been discussed, and the one on diabetes will be examined separately. Data from 20 RCTs, analyzed in a systematic review by Ferreira et al. [32], showed significant improvements in both blood pressure (BP) and the lipid profile. Specifically, decreases in systolic (SBP) and diastolic (DBP) blood pressure, total cholesterol (TC), LDL cholesterol (LDL-C), HDL cholesterol (HDL-C), and triglycerides (TG) were statistically significant when following a hypocaloric diet rich in non-oilseed legumes, but were not significant or absent in other types of hypocaloric diets—e.g., low-carb diets. Nevertheless, a few studies found that the reduction in BP and TG had more to do with the weight loss or the glycemic load rather than the legume content of the diet [52,53,54,55]. On the other hand, the majority of the included studies consistently displayed an amelioration of the plasma lipid profile with a daily integration of legumes in the diet independent of weight loss, with most data showing a reduction in TC and LDL-C [32]. These findings are corroborated by a recent review containing meta-analyses that concluded an inverse relationship between pulse consumption and TC, LDL-C, and BP. The aforementioned changes in lipid profiles have been attributed to the high content of MUFA, PUFA, and plant sterols in legumes [34]. BP is one of the most important risk factors for CVD [56]. In contrast to many meta-analyses, the most recent meta-analysis and systematic review concerning BP and non-oilseed legume intake found no significant overall effects on either SBP or DBP. However, a small non-significant decrease was noted in both. Subgroup analyses revealed a significant reduction in SBP in overweight and obese subjects [57]. Regarding the mechanism through which legumes reduce BP, most publications agree that it involves dietary fiber and plant proteins [34,57]. An earlier publication has proposed that the high content of potassium in legumes also plays a role [46]. Furthermore, bioactive peptides with angiotensin-converting enzyme (ACE) inhibitory properties have been found in fermented legumes [58]. In terms of the overall CVD risk, the protective effect of legumes as a part of diet patterns—such as the Mediterranean, DASH and plant-based diets—has been very well documented [59]. An earlier review by Marventano et al. [60] concluded that three to four servings of legumes per week significantly reduced the risk of both CVD and coronary heart disease by close to 10%. However, no association with the risk of stroke was observed. On the other hand, an analysis on the Greek sub-cohort of the European Prospective Investigation into Cancer and Nutrition (EPIC) study showed an inverse relationship between legume intake and incidence of cerebrovascular disease, such as a stroke [61]. A more recent meta-analysis of prospective cohort studies documented a reduced CVD and coronary heart disease risk with higher amounts of soy and non-soy legume intake. The risk of stroke was not statistically significant [48]. The same conclusion was reached by an umbrella review of observational studies regarding oilseed and non-oilseed legumes that found an inverse association between legume consumption and both CVD and coronary heart disease risks [62].

### 3.4. Type II Diabetes Mellitus

Extensive research has been performed when it comes to the effect of legume consumption in glucose homeostasis and T2DM patients. Reports on the association of legume intake and the risk of developing T2DM do not provide conclusive evidence. Some systematic reviews [63,64] and meta-analyses [63,64,65] showed no significant effect of legume consumption on T2DM incidence. Becerra-Tomás et al. [48] highlighted the inconsistencies through the literature and concluded that there is not enough consistent evidence pointing to a reduced risk of T2DM when intake of legumes is high. They also proposed that the heterogeneity could be attributed to the different types of legumes consumed (soy and non-soy) and the broader dietary pattern they were a part of (Mediterranean, high fiber, low GI, etc.) across the different studies. Furthermore, a recent study in a European cohort found no significant reduction in T2DM incidence rate when increasing legume intake by substituting red and processed meat in the diet [66]. On the other hand, data from the prospective observational study Prevention with Mediterranean Diet (PREDIMED) [67] on 3.349 adult subjects with a high CVD risk have shown that non-soy legumes can reduce the risk of developing T2DM when consumed in high quantities. Total legumes and lentils were inversely associated with the risk of developing T2DM. Chickpeas were also inversely associated, but with a borderline statistical significance, and dry beans along with fresh peas produced no effect. The researchers also concluded that a theoretical substitution of ½ a serving per day of eggs, rice, potatoes, whole meal, or white bread, with ½ a serving of legumes could reduce the risk of T2DM by close to 50%. This is in accordance with a review published by Ramdath et al. [68], which stated that pulses as part of a Mediterranean diet pattern can lower the risk of developing T2DM. When it comes to the effect of legumes in glycemic control, the available literature also yields mixed results. An older systematic review and meta-analysis of 41 RCTs examined the effect of non-oilseed legumes on glycemic control in adults with and without diabetes. Regarding the former, legume intake had a significant inverse relationship with fasting blood glucose and insulin. When studies with non-oilseed legumes as part of a low GI diet or a high fiber diet were meta-analyzed, a mean decrease in glycosylated hemoglobin (HbA1c) of 0.48% was noted. This reduction is significantly larger than the threshold of 0.3% considered clinically relevant by the United States Food and Drug Administration (FDA). However, in normoglycemic subjects, results were conflicting. Specifically, fasting blood glucose was reduced in studies concerning non-oilseed legumes alone, but was increased in studies with them as part of a low GI diet. These unexpected results were attributed to the high heterogeneity of the studies. The reasons could involve the different duration of the intervention and/or the follow-up period, populations characteristics, different sample sizes, methods of data collection, the study design, the types and/or quantities of legumes, and processing-cooking methods for the legumes consumed in these studies [69]. Another publication also reported an increase in fasting glucose when 64 insulin-resistant men consumed a bean-enriched low GI diet, corroborating these results [70]. A paper by Pittaway et al. [71] regarding the impact of chickpea intake for 3 months on glucose homeostasis in 45 healthy adults resulted in a decrease in fasting insulin and Homeostatic Model Assessment for Insulin Resistance (HOMA-IR). Contrary to these findings, an intervention with a hypocaloric diet rich in non-soy legumes in 42 premenopausal women with central obesity reported no beneficial effects in fasting glucose and insulin, as well as HOMA-IR [72]. Concerning patients with T2DM, a study by Jenkins et al. [73] documented a decrease of 0.5% in mean HbA1c when consuming beans, chickpeas, or lentils as a part of a low GI diet, with at least 190 g of legumes per day versus a control of high whole wheat fiber diet. One cross-over RCT on 31 patients with T2DM resulted in a reduction in fasting blood glucose and insulin after 8 weeks of replacing two servings of red meat with a cup of non-oil seed legumes for 3 days per week [45]. A recent review concludes that a mean intake of 5 cups of pulses per week consistently resulted in better long-term glycemic control, as shown by decreases of HOMA-IR, fasting blood glucose, and insulin in patients with T2DM. Moreover, it is highlighted that in acute studies involving ¾ to 1 cup of lentils or black beans, the attenuation of postprandial glucose persisted to the following meal and even the day after [68]. In terms of mechanism, the literature is in agreement and suggests that high fiber and resistant starch, low GI, plant-proteins, minerals, bioactive peptides, and phenolic compounds all contribute to antidiabetic effects [67,74].

### 3.5. Cancer

Recommendations regarding cancer prevention from WCRFI stress that it is important to include legumes—e.g., lentils and beans—in most meals, making them a core part of ones’ diet. That is mainly because they are rich in fiber, and evidence points to reducing the risk of certain tumors such as colorectal cancer [23]. Moreover, the WCFRI together with the AICR concluded after a review of the globally available literature that there is convincing evidence that foods rich in dietary fiber, such as legumes, lower the risk of colorectal cancer, overweight, and obesity, with the latter two increasing the risk for at least 12 different tumors [22]. A meta-analysis of 12 cohort studies noted a 13% lower risk of breast cancer with a higher adherence to the vegetable-fruit-soybean dietary pattern. This negative correlation was in part ascribed to soy isoflavones and a decrease in reabsorption of estrogen from the gastrointestinal tract. Furthermore, a significant inverse association among legumes and a risk of breast cancer was described in a study using data from the Nurses Health Study II. A reduction of 24% in the incidence of breast cancer was reported when consuming beans or lentils at least two times per week [75]. The relationship between soy and non-soy legume intake and risk of prostate cancer was examined in a meta-analysis of prospective cohort trials, including eight cohorts. An inverse association was found connecting high total legume consumption and risk of cancer. The dose-response analysis concluded that for every 20 g/day increase in total legume consumption, the risk of prostate cancer declined by 3.7%. In the stratified analysis, the same inverse association was observed for non-soy legumes, whereas an inverse trend was noted for soy, albeit without it reaching statistical significance [76].

Concerning the mechanism of anti-cancerogenic actions, the literature suggests that it is a result of flavonoids, mainly soy isoflavones, other antioxidants, fiber, resistant starch, phytochemicals, anti-inflammatory and anti-nutritional factors, and bioactive compounds—examples of the latter being phytic acid, tannins, saponins and protease inhibitors [76,77].

### 3.6. Gastrointestinal System

Because legumes are rich in dietary fiber, an interest has been expressed in the literature concerning potential beneficial effects on the gastrointestinal system. The latest systematic review and meta-analysis showed that there is a significant effect of legumes and their derivatives on the abundance, diversity, and richness of gut flora. However, the results were inconsistent due to a lack of in vivo human trials concerning this area [78]. A review by Knez et al. [79] focusing on the effect of fermented legumes on health and disease underlined their probiotic and antioxidant benefits. Non-soybean fermented products include basic material, lentils, beans, peas, chickpeas, and fava beans. These fermented legumes were associated with a rise in lactic acid producing a bacteria population, such as lactobacilli, which have been found to increase the bioavailability of nutrients, improve satiety, and assist in weight management. Additionally, fermented peas and beans were found to have larger amounts of polyphenols [79]. Legumes also aid in the amelioration of gastrointestinal function by increasing stool volume, regulating bowel movements, and serving as a prebiotic [80]. Nevertheless, adverse effects for the gastrointestinal tract have also been reported. Three studies [73,81,82] from the Ferreira et al. [32] systematic review reported an upset stomach, flatulence, bloating, heartburn, and diarrhea. However, their intensity and frequency were so low that they caused no dropouts in the trials where they were reported. Jenkins et al. [73] proposed that the high fiber content of legumes could be responsible for the observed gastrointestinal adverse events. Another aspect is the presence of FODMAPS (Fermentable Oligosaccharides, Disaccharides, Monosaccharides, and Polyols) in legumes which can also contribute to these adverse effects [16].

### 3.7. Depression and Anxiety Disorders

In recent years, more novel research has been performed regarding the potential effects of legumes in not-so-well-researched aspects of health. Some observational studies have indicated an inverse relationship between adherence to a Mediterranean diet and incidence of depression [83,84]. A paper by Opie et al. [85] suggests that following a diet pattern such as the Mediterranean diet and having a high intake of legumes among other food groups can help in depression prevention through antioxidant and anti-inflammatory mechanisms combined with endothelial protective properties. One study has also reported that remission of major depressive disorder is possible when adhering to a healthy diet, including legumes, based on the Mediterranean diet [12]. One randomized controlled trial with a sample size of 152 adults with diagnosed or self-reported depression focused on the outcomes of adopting a Mediterranean diet supplemented with fish oil versus a control following a regular diet. Assessments were made at baseline, 3 months, and 6 months. The analysis of the data showed a decrease of 45% in the Depression Anxiety Stress Scale (DASS), and an amelioration in all the categories of the quality of life assessment questionnaire (AQoL) regarding the Mediterranean diet group in the 3-month follow up. These results were sustained up to the 6-month mark. Specifically, higher legume intake was associated with a reduction in anxiety, stress, and negative effects, as well as an increased quality of life, coping, and psychosocial skills. The researchers highlighted a possible limitation because depression was self-reported in some participants. However, this was carried out through the DASS questionnaire, which is highly reliable, and no statistically significant difference was recorded in the baseline DASS scores between the subjects who self-reported and those that had a doctor’s diagnosis [86]. Multiple researchers stress that for normal brain structure and function, multiple nutrients are required; therefore, the quality of the whole diet rather than specific nutrients should be the focus [85,86]. A systematic review by Bayes et al. [87] has found that the polyphenol content of the Mediterranean diet could be the key factor responsible for the lower risk of depression and the amelioration of depressive symptoms.

It has to be noticed that the exact mechanism of action when it comes to legumes alone is unknown. Factors that have been proposed to mediate their effect, in the context of a healthy diet, are anti-inflammatory and antioxidant properties, along with the regulation of the endothelial function. Some authors have also briefly referred to the regulation of the microbiome-gut-brain axis [83,84,87].

### 3.8. Mortality

In regard to mortality, a study involving 7216 subjects from the PREDIMED cohort found an inverse association between cancer mortality and total legume and lentil consumption [88]. All-cause mortality was lower in the highest legume intake subgroup in a meta-analysis of prospective cohort studies, with moderate heterogeneity. No statistically significant association was made with CVD-related mortality [89]. A systematic review and meta-analysis of prospective studies examined the relationship between total protein, animal-protein, and plant-protein with mortality. Legumes were considered to be a component of plant-protein. Plant-protein intake was associated with a significantly lower risk of all-cause mortality and CVD-related mortality, but not with cancer-related mortality. In the dose-response analysis, it was revealed that for every additional 3% of energy from plant-protein, the risk of all-cause mortality dropped by 5%. The researchers concluded that substituting animal-protein with plant-protein increases longevity [90].

A groundbreaking publication by Reynolds et al. [91] examined the effect of replacing red and processed meat on several health and cost parameters. The researchers modelled five scenarios of meat replacement by well-established or novel alternative protein sources in amounts similar to these ingested at baseline. The first scenario was based on the Heart Foundation recommendations and consisted of 50 g of red meat and enough legumes, soy, seeds, and nuts to replace the rest of the intakes of red and processed meat at baseline. A scenario following the EAT-Lancet recommendation for high protein foods consisted of 14 g of red meat, and replaced the rest with poultry, eggs, legumes, nuts, and seafood. The remaining scenarios completely replaced meat intake with cellular meat, minimally-processed (MPPB), or ultra-processed (UPPB) plant-based meat substitutes. All the alternatives managed to provide better health, which was expressed as a rise in quality-adjusted life years (QALY). Most notably, the best results came from the MPPB model, whose meat alternatives were legumes and mixed meals based on legumes. The same was true for all the other outcomes measured, namely a decrease in healthcare system cost, a decrease in grocery shopping cost for each individual, and a reduction in GHG. In particular, when it came to the MPPB scenario, GHG dropped by 34%, grocery cost dropped by 7% per day, and the healthcare system savings were 5.1 billion New Zealand dollars per capita [91].

It is worth mentioning that beyond the health benefits discussed above, the effects of legumes as alternative protein sources in supplements for athletes have been examined and were very promising. Certainly, in a study conducted by Banaszek et al. where high-intensity functional training (HIFT) men and women received either whey or pea protein following an 8-week HIFT program, ingestion of either type of protein produced similar outcomes in measurements of body composition, muscle thickness, force production, work of the day performance, and strength [92]. In a meta-analysis by Messina et al. [93], results regarding the influence of total protein intake and protein source in response to resistance exercise training derived from nine studies were analyzed. It was indicated that soy protein supplementation produces similar gains in strength and lean body mass in response to resistance exercise as whey protein [93].

## 4. Legumes and Environmental Sustainability

Food systems contribute to climate change, as all stages of their production, consumption, and disposal produce anthropogenic GHG. The impact of food systems can be seen through the use of energy, land, water, and fertilizers required for food production and processing, their packaging and transport, as well as food waste [3]. On the other hand, according to the Intergovernmental Panel on Climate Change (IPCC), climate change will affect the global food supply in various ways, such as turning production to the poles, increasing the speed of plant ripeness, and thus reducing their nutrient content and altering drought and rainfall [94].

Based on self-reported dietary habits in the United Kingdom (UK), high meat consumers were responsible for 1.9 times more GHG, and moderate meat consumers were responsible for 1.5 times more GHG than people who were on a lactovo vegetarian diet (LOV), and 2.5 and 2 times more GHG, respectively, than people who consumed a strictly vegetarian diet [5]. According to a UK survey, dairy products are responsible for about 40% of GHG associated with production for LOV diets. However, when dairy is not included, as it is in vegan diets, GHG for the same kcal is much lower [95]. Beyond Meat’s Beyond Burger and Impossible Food’s Impossible Burger showed that switching from beef to either product reduces GHG, land use, and water footprints by approximately 90%. Although plant-based meat alternatives are classified as over-processed, they may exert some of the beneficial effects on CVD risk factors [25,96,97].

Additionally, waste of food is another major contributor to climate change, as the production of each kg of unconsumed food has the same environmental impact as a kg of consumed food. As with consumption, the waste resulting from PBDs has a smaller environmental footprint compared to the waste of diets with high consumption of animal products [5].

It is absolutely essential to note that it is possible to achieve satisfactory protein levels, with complete replacement of protein by plants or other sources [98]. Preferred sources of plant-based protein are quinoa, amaranth, wheat, legumes, and soy-based products such as tofu and tempeh [99]. Based on the above, protein-rich plant crops such as legumes could help reduce the need for animal sources of protein foods, which would provide huge environmental benefits.

There are various effects that legumes can have on the environment and soil quality. Leguminous plants reduce GHG by reducing mineral nitrogen fertilization, soil carbon fixation, and total mineral energy inputs to the system [100]. About 60% of N_2_O emissions come from the application of nitrogen fertilizers. It has been estimated that about 1 kg of nitrogen is emitted as N_2_O from every 100 kg of nitrogen fertilizer. The amount of N_2_O emissions depends largely on several factors, such as the rate of nitrogen application, soil organic carbon content, pH, and soil texture. In general, soil N_2_O losses from soils in legume crops are indubitably lower than those of fertilized crops, and among legumes, soybean provides the highest protein (g) per GHG compared to other plant and animal protein sources [101].

Leguminous plants can be included in various types of cultivation; e.g., in rotation and intercropping. Their inclusion in such systems helps to maintain soil fertility by providing nitrogen through biological nitrogen fixation (BNF), and increasing soil organic matter and resources available for soil heterotrophic organisms [101,102,103]. This process of BNF requires a symbiotic relationship between bacteria called rhizobia and legumes. Legumes and other leguminous plants’ crops fix nitrogen in the soil, which is then converted to NH_3_ by the action of rhizobia, and eventually is absorbed by the crops to form proteins. This process helps the soil maintain its fertility and improve the yields of the next crop [103]. In addition, although cereal leguminous plants are weak suppressors of weeds, mixing species in the same cropping system appears to ameliorate the ability of the crop itself to suppress weeds [104]. Furthermore, by intercropping cereals with legumes, cereals—while benefiting from legume-bound nitrogen—increase the bioavailability of iron and zinc in the accompanying legumes [105].

Another advantage of legumes’ crops is their resistance to environmental threats. According to archaeological research, lathuri is one of the first legumes cultivated by man. It is a plant with excellent agronomic properties, such as drought, flood and salinity resistance, high nitrogen regulation capacity, easy cultivation with low need for fertilizers, and adaptability to different climates and soils [106]. It is a crop that survives during severe droughts, and its consumption is the only solution for many low-income societies. Thousands of people have survived famines thanks to lathuri. It can contribute as a source of multiple stress-resistant genes for the genetic improvement of crops. The basis of increased drought and salinity tolerance should be studied so that the crop can be used as a resource of germplasm for traits to adapt the most important crops to new conditions [107].

Lupin is considered as an optimal alternative to soybean cultivation in cold climates, and constitutes a choice crop for cultivation in Al-rich acidic soils in temperate climate regions [108]. The biological value of lupin protein is comparable to that of soy, and contains essential amino acids such as lysine, leucine, and threonine. Lupin is an economically and agriculturally advantageous plant, can stand as an adequate alternative to soybean, and is therefore suitable for sustainable production [109]. However, it has to be taken into consideration that problems associated with the formation of acrylamide can arise when lupin flour is used for the production of cereal products [110].

Soybeans are one of the most valuable crops in the world because of their multiple uses, both as an economical source of protein, healthy unsaturated fats, and carbohydrates for human consumption, and as animal feed. It is by far the cheapest source of protein for poor smallholder farmers, compared to other protein-rich foods such as animal meat, fish, eggs, and milk. In terms of protein quality, the protein value of soybean is similar to that of eggs [111]. Soybean produces the highest amount of protein per hectare and is able to meet future global protein needs. Globally, soybean production is projected to increase by 371.3 million t in 2030 [112]. Conventional protein sources are highly expensive, making them inaccessible to a vulnerable population. Therefore, the development and production of soy-based protein foods are an important solution to malnutrition and hunger.

Although the benefits of consumption and use of legumes are supported by a large body of evidence, concerns regarding their nutritional value in comparison to animal-protein sources have to be taken into consideration. Proteins from animal sources are different from those of plant sources, and there is a great variation in amino acid composition as well as in protein digestibility. Among legumes, soy is the one which contains all essential amino acids (EAAs) in adequate proportions to cover human body needs. It is comparable to dairy, egg, and meat, which are considered complete sources of protein. Compared to animal sources, legume sources generally have lower levels of sulphur amino acids—i.e., methionine and cysteine. In addition, as has been referred to before, legumes contain anti-nutritional factors which affect protein digestibility. Trypsin inhibitors inhibit digestion in the small intestine, and tannins bind proteins, making them unavailable for digestion [113]. Where EAAs are lacking, complementary protein sources should be combined in order to ensure the quality of protein. In several cases, a complete substitution of animal proteins with plant-based foods needs food supplements to be included in the diet. In a systematic review by Neufingerl and Eilander [114], the results of observational and intervention studies conducted to assess nutrient intake and status of adult populations consuming plant-based diets and that of meat-eaters were analyzed. It was found that both groups had dietary inadequacies in the intake and status of nutrients which were mainly present or were more bioavailable in the other group. Certainly, plant-based dietary patterns were found to increase the risk of inadequate intake and status of EPA/DHA, vitamin B12, D, iodine, iron, zinc, and calcium, while in the case of meat-eaters, an increased risk was found for nutrients that are more present in plant foods; i.e., fiber, PUFA, ALA, vitamin E, folate, and magnesium [114].

### Perspectives for the Future: Viability of Legumes on Mars

While it is known that Martian soil contains the majority of the macronutrients and micronutrients necessary for plant growth, it also contains some factors that potentially limit growth, such as high salinity, perchlorates, and sulfates [115]. Over the years, various ground simulators have been developed to simulate Martian regolith for future mission development and scientific and engineering research. Several studies have reported that these regolith simulants have supported the growth of various crops [116,117]. For example, it has been reported that plant growth was possible in Johnson Space Center Mars-1A regolith simulant without any addition of nutrients. In this study, plants such as tomato, wheat, and cress performed particularly well [118].

The presence of about 2.7% nitrogen in the Martian atmosphere and the “bound” nitrogen in the Martian soil offers the potential to exploit one of the most important plant-microbe compounds on Earth. Legumes can be used to enrich Martian soils with nitrogen, which will be useful for non-legume plants that are not as efficient at nitrogen uptake. According to a study investigating the establishment of legume-rhizobia symbiosis in different Martian soil simulations, it was found that legume-rhizobia symbiosis can be supported [119].

It is worth noting that the studies are carried out on discs, not in full soil culture, and in atmospheric conditions that do not reflect the conditions of the planet in question. For example, the lower amount of nitrogen in the atmosphere of Mars compared to Earth may affect the ability of legumes to fix atmospheric nitrogen. Future studies may investigate the symbiosis of legumes and rhizobia in atmospheric conditions closer to Mars. However, in all likelihood, all plants will be grown on Mars in greenhouse conditions with enhanced atmosphere, supplemental lighting, and moderate temperatures.

## 5. Legumes in the Food Industry

Legumes are safe for consumption, relatively cheap, and readily available. Thanks to their nutritional and physicochemical properties, they are widely used by the food industry to develop products for the general population, but also for specific groups such as vegetarians, T2DM, and celiac disease patients [120].

Legumes have been incorporated into new product formulations, capitalizing on the trend of plant protein. For instance, legume-based pasta, breads, and snacks are being made, demonstrating the food industry’s willingness to incorporate legumes to meet consumer needs and demands [49]. For consumers in Europe, the demand for legume-based products is mainly driven by the awareness regarding the health benefits of legumes. There is also a strong market trend for gluten-free products both in the EU and the US, stimulating a demand for legume-based flours [121]. Accordingly, for the Australian market, a survey identified 300 new products launched between 2012–2017 that contained either vegetables and legumes or legumes alone, with at least half a portion (1 portion = 75 g) of legumes appearing as the main ingredient [122].

The protein components of legumes are increasingly being used as a viable alternative to animal proteins. For example, lentil proteins have been used in the production of doughnuts, cakes, and crackers enriched with protein or that are gluten-free. In addition, together with transglutaminase, lentils have been used to make protein-enriched steak or burger substitutes, and as an emulsifier for salad dressing [123,124]. They have also been used as stabilizers in nano-emulsion systems, in omega-3 fatty acid supplement capsules, as components of antimicrobial membranes, and in the production of nanofibers [124]. Due to the fact that lentil is a crop that expands rapidly, since it can be used for direct human consumption, it can have an impact as a protein source for food processing applications. Similarly, pea proteins have been used in the production of many meat substitutes because of their functional properties (including solubility, water retention capacity, oil retention capacity, emulsifying properties, foaming properties, and gelling properties) that contribute to the structure and texture of foods. Also, compared to soy protein, pea protein is generally recognized as a non-food allergen with a relatively high nutritional value and without genetic modification, offering a clean label for food products [125]. However, it has to be taken into consideration that pea-protein is not an-allergic, since anaphylaxis to hidden pea protein has been reported [126]. It also has to be mentioned that although pea protein is an excellent ingredient for improving the nutritional and technological properties of foods, it forms weaker and less elastic gels than soybean protein during food processing. In addition, further studies are needed in order to improve the organoleptic characteristics of foods containing pea protein, especially the undesirable flavor and color. Chickpeas are used to produce chickpea flour, which can be used as a substitute for common flour for the development of food products such as noodles, breads, cookies, and sausages [127]. Chickpeas as an alternative protein source enhance the nutritional value of foods due to adequate levels of essential amino acids content and digestibility. Their organoleptic characteristics, such as the light color, the neutral taste, and the bland flavor, make them appropriate for applications in the food industry.

In addition to edible oil, soybean is processed to give foods such as soy germ, toasted soy protein flours, soy milk, tofu, tempeh, miso, natto, soy paste, and soy sauce, as well as bean curd, oncom, tauco, ice cream, and soy flour with different techniques, including dehulling, flaking, and defatting [111,128]. Since lupin is rich in protein, lupin flour could be used as an excellent ingredient to enhance various foods, and can replace egg in cakes, pancakes, cookies, pasta, or bread [129]. It can also be incorporated into wheat flour to enhance the nutritional value of the final products, as it is rich in lysine and low in methionine and cysteine, whereas wheat flour is poor in lysine and rich in sulphur amino acids. Lupin protein powder can also be blended with fruit juice, smoothies, or added to breakfast muesli or soups. Lupin products, such as flour and isolated and concentrated proteins, are commonly used in bakery products and in the production of gluten-free products as secondary ingredients.

As was discussed earlier, legumes contain anti-nutrients such as tannins, lectins, phytic acid, and oligosaccharides, which affect the digestibility and bioavailability of nutrients. The amount of anti-nutrients may be reduced by food processing. Several pre-treatments, including fermentation with lactic acid and yeasts, are used to improve the nutritional and organoleptic profile of legume products, increasing their acceptability to the consumer [50,130].

### 5.1. Legume-Based Alternatives to Animal Products

The consumption of plant-based alternative foods (PBAF), although still relatively small as a percentage of daily dietary energy intake, has increased significantly and appears to be accelerating. Consumption is higher among those who eat less meat, supporting the hypothesis that these products have a role in the dietary transition from animal products [9]. Sensory properties (positively hedonic plant-based products as animal-based foods) and consumer food consciousness are important factors related to the development of PBAF [131]. High variation is observed in the adoption of PBAF among consumers and among different types of products. An interesting study which took place in Sweden [132] and investigated consumer attitudes and beliefs on three different types of plant-based meat alternatives—i.e., two highly processed plant-based meat alternative products and pulses (pre-cooked beans)—showed that such products were perceived by the volunteers as more modern, artificial, and expensive compared to pulses, which were thought to be healthier and a better choice for the climate. Another study that was conducted on a sample of Danish consumers revealed that those with more frequent intake of meat (or of all animal products) expressed negative attitudes about protein content, satiety effect, taste, environmental effects, and health effects which could serve as barriers towards adopting a plant-based diet. On the other hand, consumers with lower meat intake expressed positive attitudes towards the ease of cooking, taste, protein content, satiety effect, and availability of plant-based food. Such attitudes could serve as facilitators for the adoption of a more plant-based diet [133]. The most common barrier for consumers is the difficulty in preparing and finding available options for meatless meals [133,134,135]. Other barriers arise from the fact that meat consumption is a habit [134,135], and that it is considered a necessary component of a “proper meal” [135]. In addition, there is a lack of knowledge regarding the negative effects of high meat consumption on the health and environment, and negative perception and concerns about high levels of sodium as well as the high degree of processing of replacement products. Even though large prospective studies and meta-analyses have shown that total mortality rates are modestly higher in subjects consuming high intakes of red and processed meat than in those with low intakes, for poultry, no or moderate inverse associations have been reported [136]. Nevertheless, meat is a good source of energy, protein, and micronutrients such as iron, zinc, and vitamin B12. As was referred to before, plant-based dietary patterns increase the risk of inadequate intake of such nutrients, and this may have negative health impacts. When excluding meat from the diet, sufficient intake of nutrients which are mainly found in meat and meat products is possible; however, a variety of other food products should be available and consumed. This is not always a given, especially in low-income countries [136]. According to the World Cancer Research Fund (WCRF), people who eat red meat are recommended to consume amounts less than 500 g a week, and the population’s average consumption should not exceed 300 g a week. The fraction of processed meat should be minimized in any case [137]. Finally, other barriers in adopting a plant-based diet coming from social norms like reactions from friends and family, or stigmatization of vegetarians and vegans according to some studies [134,135]. However, this phenomenon has not been observed in other studies [135,138,139].

Research on the environmental impact of PBAF compared to their animal-based counterparts has shown promising results in terms of GHG, land use, and blue water footprint, and suggests that they could play a key role in mitigating climate change through the food system [140,141]. A major advantage of the industrial production of PBAF—for instance, compared to poultry—is the possibility to enrich them with micronutrients. This could benefit populations in developing countries, where nutritional deficiencies are widespread [142]. However, the environmental impacts of plant-based meat alternatives need to be assessed. There are studies showing that such products are more sustainable than meat products; however, they are highly processed foods, and in this context, the use of different forms of energy for the production of plant-based ingredients counteract the low environmental impact [141,143]. In terms of the health effects of PBAF, it has to be noted that there are studies showing that plant-based products are not healthier than animal products [144]. Although plant-based meat alternatives generally have lower calories and a lower fat content, they have higher amounts of carbohydrates and higher levels of sodium [141,145]. A high degree of processing and heterogeneity in formulation, as well as differences in digestibility and bioavailability between proteins from animal and plant origin cause skepticism and questioning regarding the unknown effects of plant-based meat alternatives on human health [144].

Some examples of PBAF based on legumes are as follows.

### 5.2. Vegetable Cheese Analogue

Among dairy alternatives, cheese remains the biggest obstacle for people interested in switching to a vegan diet. The plant-based cheese alternatives (PBCAs) industry has not yet been able to replicate the melting and elasticity of cheese, and most PBCAs on the market have a chalky, pasty, and plastic-like texture. Plant proteins have a higher molecular weight and different functional properties than milk, and it is therefore difficult to mimic the texture of cheese. The easiest cheeses to mimic are those with a spreadable and creamy texture, such as feta, ricotta, or cottage cheese, and those with a strong flavor, such as spicy and smoked products, which mask the taste of the plant source [146].

Another disadvantage of PBCAs is that most of them are mainly based on coconut oil. Hence, they have a lower nutritional value, lower calcium, and lower protein content than conventional animal cheeses. In general, 50% of PBCAs contain little to no protein (<0.5%). This does not satisfy consumers who, today, are more aware of the negative health effects of processed foods. Still, they are concerned about the protein content of foods and are attracted to products made from legumes or nuts that are fortified with calcium and vitamin B12 [147]. Therefore, the development of alternative cheese products with a comparable protein content to animal cheese would be a significant advance in this field. Legumes could be more suitable than any other plant ingredient for PBCAs. The main reasons are their high protein content, almost double that of whole and pseudo-grain cereals, and their low cost compared to that of nuts. However, in general, all PBCAs are more expensive than cow’s cheese. More often than not, the price of a PBCA made from legumes does not reflect the price of its ingredients, which are usually cheaper than dairy ingredients. This is because it is an innovative product produced on a small scale, and its marketing is limited to the vegan consumer. It is therefore argued that legume-based products should not be placed in the vegan section of supermarkets, which is visited by only this consumer group, but should be marketed to all health-conscious consumers [147].

### 5.3. Infant Formulas

The specific needs of certain population groups, including infants, should be taken into account when designing interventions on climate change and healthy eating. Children are the part of the population most vulnerable to the effects of climate change. Food security for infants and young children is not possible without promoting and achieving high rates of breastfeeding, goals which have never been reached on a global scale [148,149]. The alternative to breastfeeding is infant formula, which is usually based on cow’s milk.

There are only a few commercially available plant-based infant formulas containing either soy or rice protein. In the European Union, the only source of protein allowed in infant formula is cow’s milk, goat’s milk, soya, and hydrolyzed proteins. Soy-based infant formulas were introduced almost 100 years ago, although it has undergone changes to achieve higher digestibility and lower dietary fiber and vegetable salt content. In addition, they have been fortified with the amino acids methionine, taurine, and carnitine, as well as choline and inositol, and more recently, they have been supplemented with long-chain polyunsaturated fatty acids (LCPUFAs) [150]. Unlike soy beverages that use raw soy as a base ingredient, the infant formula uses an isolated soy protein to produce a high purity protein product containing at least 90% protein. Also, heat treatment and extraction during the processes reduce certain anti-nutrients [151,152].

Meta-analyses on the safety of soya-based infant formula (SIF) have shown that the administration of SIF to normal term infants is associated with normal growth, adequate protein intake, normal bone formation, and normal immune development. According to the few studies that have evaluated the impact of SIF on neurodevelopment, no differences in IQ, behavioral problems, learning disorders, or emotional problems have been found in school-age children fed either SIF or breastfeeding during the first year of life. In addition, no differences have been found between males and females in educational attainment either when they were fed SIF or formula-fed as infants [151]. Soy can be safely incorporated into children’s diets under the principles of variety and moderation [153].

Soy allergy is less common than cow’s milk allergy, although it affects about 0.3–0.4% of young children [154]. Because of the perceptible nutritional disadvantages and the potential for causing an allergic reaction, the European Society for Paediatric Gastroenterology Hepatology and Nutrition (ESPHGAN), European Academy of Allergy and Clinical Immunology (EAACI), and American Academy of Pediatrics (AAP) do not recommend the administration of SIF to infants under 6 months of age. However, ESPGHAN and AAP state that SIF may be considered in infants over 6 months of age when complementary feeding has been initiated and in the absence of soy allergy for infants with cow’s milk allergies and when parents wish to exclude products of animal origin [155]. Despite recent statements by experts stating that SIF is safe, affordable, and an alternative to cow’s milk-based formula for term infants, its popularity is declining. On the contrary, hydrolyzed rice-based infant formula is increasingly being used, particularly in infants with functional gastrointestinal disorders or when a cow’s milk allergy possibly exists [152].

Pea protein is also used in infant formulas. It is highly soluble and therefore easily digestible and easily absorbed. The Protein Digestibility Corrected Amino Acid Score (PDCAAS) of pea protein isolate is 89%, contrary to that of soybean, which is 92% [152,156]. It is rich in branched chain amino acids, arginine, and lysine. Pea flour has been used as base for infant formula and documented in research since 1953 [157]. Infant formulas with partial substitution (50%) of dairy proteins with pea and faba bean proteins have been developed and examined regarding the in vitro protein digestibility and PDCAAS. The results of this study showed that faba bean proteins are a promising ingredient for the partial substitution of whey proteins. However, in vivo studies have to be conducted in order to confirm these results [158].

### 5.4. Aquafaba

Aquafaba, which is the liquid resulting from the boiling of chickpeas, was found to be a suitable alternative emulsifier for the production of vegan egg-free emulsions. The aquafaba-based emulsions, containing mixtures of proteins with different cold-pressed oils, had higher radical species scavenging activity, better oxidative stability, and similar color parameters compared to commercial vegan mayonnaise and to traditional emulsions containing egg yolk as the main emulsifier. The new plant-based emulsions could be a promising alternative to mayonnaise and egg-based salad dressings [159].

### 5.5. Legume-Based Beverages

Although plant-based beverages can be produced from various ingredients such as dry nuts, fruits, and cereals, they are not considered good substitutes for milk, as they are poor in protein. Legume-based beverages exert the most balanced composition, and their protein content is similar to that of cow milk. The most widely consumed legume-based milk substitute is soy drink. However, its demand has begun to wane since questions were raised regarding genetically modified foods, allergies, high levels of isoflavones, and its carbon footprint. Other legumes that have been used to produce milk substitutes are peas, lupine, lentils, chickpeas, and beans.

Although the nutritional composition of legumes is appropriate, technological issues concerning processing, preservation [160], and organoleptic characteristics need to be addressed. In a study by Aydar et al., plant beverages were produced based on two different varieties of red beans. Although these drinks were high in protein and antioxidants and had a better fatty acid profile compared to commercial plant beverages, they had a very strong bean flavor. For this reason, they did not enjoy the most general acceptance compared to commercial beverages [161]. In another study, lactic acid bacteria were used to ferment water beverages based on legumes, and then their effect on the organoleptic characteristics and their protein content was examined. After fermentation, lupine- and pea-based beverages presented better organoleptic properties and continued to be high in protein [162].

Plant-based yogurt alternatives which are currently found in the market are mainly based on coconut or soy. Coconut preparations are high in saturated fat and low in protein. Soy raised concerns to consumers for the reasons which were referred to before. Due to their high protein content, their ability to formulate gels, and fermentation by lactobacilli cultures, pulses seem to be an appropriate basic ingredient for making a yogurt alternative. Yogurt alternatives can be prepared either by using whole pulses or isolated pulse proteins [163].

## 6. Supporting the Production and Consumption of Legumes

The anticipated future higher demand for food will require not only larger stretches of land under cultivation and increased yields, but more worryingly, according to predictions based on the current operation of the industry, greater livestock production. Indeed, recent predictions confirm that world meat consumption will increase by 76% by mid-century [136]. This means that, over time, unless consumption patterns change, pressure will increase on the earth’s limited resources, as livestock production requires the use of natural resources and produces unwanted by-products that increase environmental degradation [3].

The evolution of agricultural practices has been based on the adoption of the most widely used and profitable techniques, as well as the most lucrative crops. This has led to a favoring of certain crops and the marginalization of other less profitable species. As a result, producers have turned to more profitable crops, such as cereals, at the expense of other crops, such as pulses. The promotion and use of agrochemicals became the main standard, completely ignoring the potential risks to the environment [164]. The low profit for farmers from pulses is probably due to the underestimation of their benefits, the lack of interest from the agro-industrial supply chain that undermines the additive value of pulses, and the low profits caused by insufficient compensation for the relative reduction in the use of artificial fertilizers. Food sustainability experts and policy makers need to work more closely with farmers and the food industry to ensure that these supply chains are working towards social, economic, and environmental sustainability, which should be encouraged by government interventions.

On the other hand, the public needs to be informed about specific plant-based food sources and reassured that their protein needs can be adequately met. One possible strategy to alleviate health concerns is to provide appropriate nutrition education to medical students and health professionals, as physicians often lack important nutrition knowledge and counselling skills needed to successfully guide their patients [25]. In order to reach a consumer behavior change, affordability and palatability should be provided, and benefits for health should be made clear, raising awareness and shifting standards so that plant-based foods and specifically legumes and legume-based products become the default choice [165].

Regarding the limited availability of legume-based options outside the home, new policies could be implemented imposing healthy legume-based options on canteens in schools, hospitals, universities, and other government agencies in order to reduce the accessibility barrier. In addition, more information and knowledge about food should be available to encourage better consumer choices and raise awareness of their consequences: for example, communicating the environmental footprint to consumers through labels or raising consumer awareness of food-related emissions. Furthermore, providing a predetermined choice of sustainable foods at various events or venues seems to be significantly effective in helping consumers to choose sustainable foods [166]. Progress has already been made with restrictions on junk food advertising, and there has been a widespread change in recent years with grocery stores offering significantly more plant-based product choices than in the past [167].

The use of legumes as a sustainable source of protein has the potential to create new markets, reduce healthcare costs, and create new job opportunities. However, there could also be economic challenges associated with this transition, such as the need for improved infrastructure and potential job losses in other fields. Finally, the economic impact of using legumes as a major source of protein will depend on many factors, including market demand, technological developments, and government policies.

There is no common decision on the recommended portion size of legumes in a balanced diet, which prevents the development of awareness strategies to increase consumption. In a variety of legumes, 100 g of cooked legumes are in line with most local portion sizes for legumes and provide significant levels of nutrients that are under-consumed by specific age and gender groups. In addition, this quantity provides a range of nutrients that qualify for nutrient content claims under regional regulatory frameworks. It is noteworthy that in regions where legumes are widely consumed and are a staple food, 100 g of legumes can be easily consumed daily. However, in areas where a wide variety of foods are available, this amount should be increased at the expense of other foods [168].

**Figure 1:**
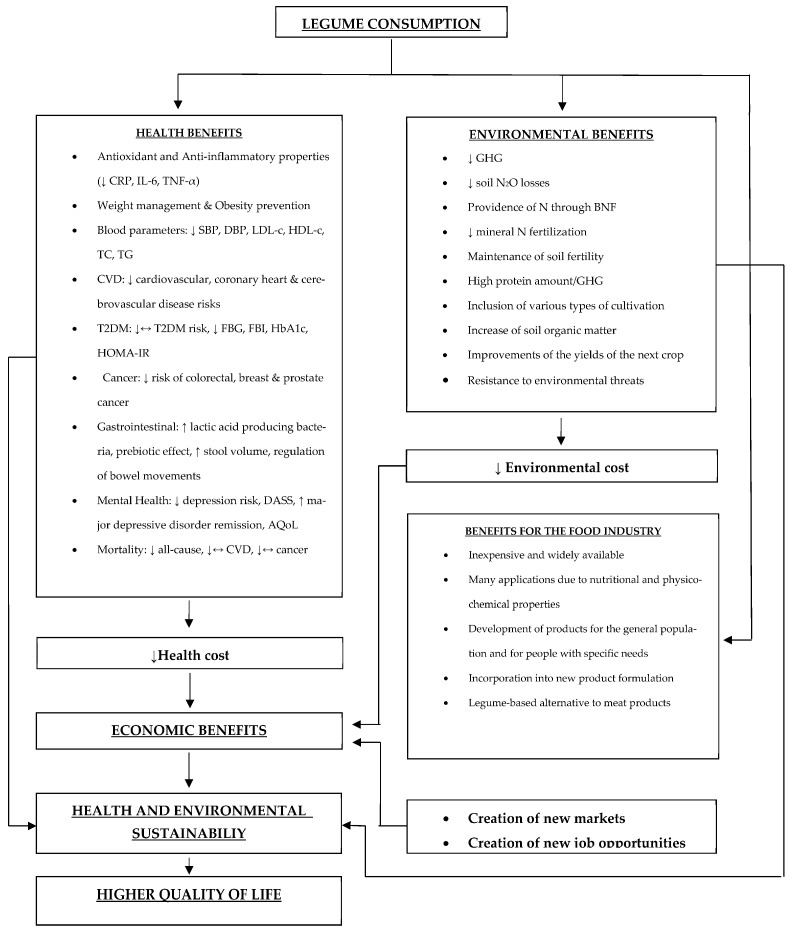
Synthesis diagram illustrating health, environmental benefits, and subsequent economic benefits of legumes.

Legumes can produce many health benefits, therefore increasing good quality life expectancy and reducing healthcare costs. They are also inexpensive and widely available, which results in a measurable reduction in daily grocery shopping costs [91]. They exert many benefits in multiple areas of health, including overweight and obesity, the blood lipid profile, blood pressure, cardiovascular diseases, diabetes, several cancers, gut health, and mental health, as indicated in this paper. Therefore, they are an asset in improving and preserving public health and longevity. Moreover, they lower GHG and are thus ecologically sustainable. Another aspect of sustainability could be the cost to the individual as well as to the public health system, and as previously mentioned, legumes reduce both, being also economically sustainable [169] (Figure 1).

As the European Commission stresses, it is necessary to implement changes in several food-related areas, such as education, research, innovation, funding, and corporate social responsibility. More integrated food policies are required, and there is a need for multi-level governance that promotes cooperation and exchange of practices, as well as further support for inclusive initiatives. Researchers and practitioners in the field of sustainable food systems need to collaborate and partner with food and agriculture industries, health and social services, educational institutions, as well as policy makers, the media, and civil society.

### Study Limitation

A limitation of the present review is that due to the broadness of the theme, it was not possible to cover all the aspects regarding the effects of legumes on health and environmental sustainability, and this may restrict the information presented. Another limitation is that environmental attitudes, consumers’ motives which drive their interest on food products, and associative networks affecting communications of different perspectives of food products under debate such as plant-based alternatives to meat, are not discussed in the present study. This understanding could help the food producers address consumers’ motives and demands, and proceed to appropriate marketing activities [170]. In addition, studies regarding the economic impact of legume consumption are not discussed in this paper.

## 7. Conclusions

Today, legumes can play an important role in peoples’ diets for many reasons. Firstly, thanks to their nutritional content, they have been associated with good health and longevity. They are recognized as excellent sources of protein, complex carbohydrates such as starch and dietary fiber, vitamins, and minerals in the human diet. The presence of bioactive components in legumes has been shown to favor the prevention of chronic diseases including T2DM, CVD, inflammatory processes, and carcinogenic processes. Legumes can be grown in a variety of climates and soil types and require less water and fertilizer than other crops since they have the ability to capture nitrogen from the atmosphere, making them a sustainable option for farmers. Moreover, they constitute an inexpensive source of protein, so they are accessible to people of all income levels. It is also a versatile ingredient that can be used in many different recipes. Legumes such as soybeans, lentils, and chickpeas can be processed to create a texture and taste similar to that of meat. This can provide a viable and plant-based source of protein for people who want to reduce their meat consumption.

However, it has to be mentioned that multiple research articles throughout the literature assess the impact of legumes on the health and environment in the context of a broader dietary pattern—e.g., Mediterranean, low GI, high fiber diets, or plant-based diets—thereby limiting the ability to separate the impact of legumes from the effect of the whole diet. On the other hand, this could be seen as an asset, because in actuality, legumes are consumed as part of a broader wholesome diet. Future studies have to be carefully designed and conducted in order to answer questions regarding the health, the environmental impact, and the economic impact of legumes and legume-based food alternatives compared to other meat alternatives and meat products.

The presented findings support the claim that legumes could be an excellent component of sustainable health and environmental practices. Achieving widespread adoption of legumes as a sustainable source of protein will require a miscellaneous approach, including education, research and development, policy changes, and collaboration between different interested parties. By joining forces to accomplish this goal, we can promote sustainability, health, and nutrition for both our generation and future generations.

## Figures and Tables

**Table 1:** Nutritional composition of common legumes.

	Navy Beans	White Beans	Broadbeans/ Fava Beans	Chickpeas	Lentils	Peanuts	Peas	Soybeans
Energy (kcal)	140	139	110	164	116	567	118	172
Protein (g)	8.23	9.73	7.6	8.86	9.02	25.8	8.34	18.2
Total fat (g)	0.62	0.35	0.4	2.59	0.38	49.2	0.39	8.97
Carbohydrates (g)	26	25.1	19.6	27.4	20.1	16.1	21.1	8.36
Dietary fiber (g)	10.5	6.3	5.4	7.6	7.9	8.5	8.3	6
Iron (mg)	2.36	3.7	1.5	2.89	3.33	4.58	1.29	5.14
Calcium (mg)	69	90	36	49	19	92	14	102
Magnesium (mg)	53	63	43	48	36	168	36	86
Phosphorus (mg)	144	113	125	168	180	376	99	245
Potassium (mg)	389	561	268	291	369	705	362	515
Sodium (mg)	0	6	5	7	2	18	2	1
Zinc (mg)	1.03	1.38	1.01	1.53	1.27	3.27	1	1.15
Ascorbic acid (mg)	0.9	0	0.3	1.3	1.5	0	0.4	1.7
Folate DFE (μg)	140	81	104	172	181	240	65	54
Cholesterol (mg)	-	0	0	0	0	0	0	0
Saturated fatty acids (g)	0.10	0.09	0.07	0.27	0.05	6.28	0.05	1.3
Monounsaturated fatty acids (g)	0.14	0.03	0.08	0.58	0.06	24.4	0.08	1.98
Polyunsaturated fatty acids (g)	0.49	0.15	0.16	1.16	0.17	15.6	0.16	5.06

All values are referring to 100 g of mature seeds, cooked—boiled, without salt—except for peanuts, which are referring to a 100 g of raw peanuts. DFE: Dietary folate equivalent [31].

## Data Availability

Not applicable.
